# Correlation between connexin and traumatic brain injury in patients

**DOI:** 10.1002/brb3.770

**Published:** 2017-08-09

**Authors:** Bonian Chen, Liwei Sun, Xiaozhe Wu, Jun Ma

**Affiliations:** ^1^ Tianjin Huanhu Hospital Tianjin China; ^2^ School of Public Health Tianjin Medical University Tianjin China

**Keywords:** brain surgery, connexin, CX43 phosphorylation, neuronal injury, traumatic brain injury

## Abstract

**Background:**

Identification of molecular alterations of damaged tissue in patients with neurological disorders can provide novel insight and potential therapeutic target for treatment of the diseases. It has been suggested by animal studies that connexins (CXs), a family of gap junction proteins, could contribute to neuronal cell death and associate with neurological deficits during trauma‐induced damage. Nevertheless, whether specific CXs are involved in traumatic brain injury (TBI) has remained unexplored in human patients.

**Methods:**

In a clinical setting, we performed a correlation study of 131 TBI patients who received brain surgery. CXs (including CX40, CX43, and CX45) were examined in the harvested brain tissues for studying the relationships with the Glasgow Coma Scale scores of the patients.

**Results:**

Specifically, the protein levels of CX43 (negatively) and CX40 (positively) are associated with the extent of disease severity. Meanwhile, the phosphorylation status of CX43 was strongly associated with the severe TBI patients who contain relatively high kinase activities of PKC (protein kinase C) and MAPK (mitogen‐activated protein kinase), two possible activators for CX43 phosphorylation.

**Conclusion:**

These data highlight that a cluster of connexin family gap junction proteins not previously studied in humans is significantly correlated with the disease progression of TBI.

## 1 INTRODUCTION

Traumatic brain injury (TBI) is a global health problem, with an estimated 10 million patients affected annually around the world (Hyder, Wunderlich, Puvanachandra, Gururaj, & Kobusingye, 2007). The negative impacts of trauma could last for an extremely long time and have latent effects on the central nervous system. As the serious complications of TBI, including a loss of memory or impaired cognition, are not often easily discernible, early identification of risk factors for severe consequences of TBI patients is a priority for public health (Ghajar, 2000). On top of that, due to the lack of standard method to quantify the magnitude of acute brain injuries, outcome prediction for established TBI patients is usually hard, leading to missed opportunity for appropriate and sufficient treatment and care (Bruns & Hauser, 2003; Dewan, Mummareddy, Wellons, & Bonfield, 2016; Ghajar, 2000; Thurman, 2016). As a result, increasing numbers of TBI patients in children and elderly people are coupled with high risks of either mortality or prolonged hospitalization (Dewan et al., 2016; Korley, Kelen, Jones, & Diaz‐Arrastia, 2015; Thurman, 2016). Dissecting the molecular alterations of brain tissues following TBI could potentially identify therapeutic targets. It may also facilitate the delineation of the neuron cell damage that helps brain injury diagnosis, therefore improving the care management of TBI patients at high risk of severely adverse symptoms in clinic.

As secondary neuronal degeneration that contributes to TBI could be resulted from the transmission of damage signals produced in the injured cells, the component of direct cell–cell contact such as gap junction may play key roles in the pathogenesis of TBI. Connexins (CXs) are four‐pass transmembrane proteins belonging to gap junction protein family (Nagy, Dudek, & Rash, 2004; Schulz et al., 2015). The expression of these proteins in the mammalian central nervous system is believed to participate in neuron cell communications through electrical synapses, which appears to be involved in neuronal injury such as ischemia, epilepsy, spinal cord injury, and TBI (Nagy & Rash, 2000; Perez Velazquez, Frantseva, & Naus, 2003; Talhouk, Zeinieh, Mikati, & El‐Sabban, 2008). Particularly, in vitro trauma‐induced cell death and impairment of synaptic function are both reduced in organotypic hippocampal slices from CX43 (*GJA1*, gap junction protein alpha 1 or connexin 43) knockout animals (Frantseva et al., 2002). A CX43 blocker that decreases CX43 protein results in reduced inflammation and tissue damage and improves hind limb function in an in vivo model of spinal cord injury (O'Carroll, Gorrie, Velamoor, Green, & Nicholson, 2013). The serum level of CX43 was implicated as a biomarker at various postinjury time points in a mouse model (Ahmed, Plantman, Cernak, & Agoston, 2015). In addition, other CXs were also found to be expressed in the astrocytes or microglia localized in the injury sites of the brain, and their levels are altered as well during the development of brain injury in animals (Lin et al., 2002; Moon, Choi, Kim, Kim, & Sun, 2010; Ohsumi et al., 2006; Xie, Cui, Deng, & Feng, 2015). Together, these preclinical studies have suggested that the CXs could contribute to the propagations of injury‐related signals and thus potentially correlate with the pathological status of the CNS injury in TBI. Until now, this hypothesis has not yet been studied in human patients. It also remains unknown if specific CXs could be used as predictive, diagnostic, or prognostic markers for TBI. By examining damaged brain tissues, we sought to take an early step in studying the potential relationship between the levels of specific CXs and the injury severity of TBI patients. As CX43 phosphorylation has been specifically associated with brain injury and regulated by protein kinases (Schulz et al., 2015), we also compared the levels of CX43 phosphorylation and activities of relevant kinases between the selected TBI patient groups. The results may provide further human evidence to suggest connexin proteins as essential players involved in TBI‐related neuronal deficits.

## 2 MATERIALS AND METHODS

### 2.1 Patient cohorts and study samples

This study was approved by the ethics committee of Tianjin Huanhu Hospital and informed consent forms were obtained. Impaired cortex was obtained from TBI patients during brain surgeries within 8 hr after injury, which was snap‐frozen during the collections. The experimental analysis was performed on the consenting patients who were enrolled into Tianjin Huanhu Hospital, and is in compliance with the guideline approved by the Tianjin Huanhu Hospital. The GCS (Glasgow Coma Scale) score system was used to evaluate the severity of TBI during hospitalization. Three patient groups were then determined with the assessment of consciousness as follows: moderate group with GCS 10–12, severe group with GCS 6–9, and extremely severe group with 3–5. The medical history of all subjects was also reviewed for inclusion/exclusion criteria. Inclusion criteria include: (1) clear indication for surgery of craniotomy with imaging diagnosis by computed tomography scan; (2) reported incidence of traumatic head injury accompanied by brain contusion; (3) age from 18 to 60, with education levels above middle school. Exclusion criteria include: (1) preexisting history of TBI and cerebral vascular accidents; (2) preinjury history of drug abuse or long‐term heavy alcohol consumption; (3) mental illness, or other intracranial diseases such as encephalitis, etc., other dementia‐related diseases before injury; (4) severe multiple injury associated with other organs. Total of 174 consenting patients were recruited. Forty‐three patients were excluded and 28 patients were withdrawn during the study.

### 2.2 RNA extraction and real‐time PCR

In order to assess the expression levels of connexin transcripts, total RNA was isolated from the harvested tissues using the RNeasy kit (Qiagen, Valencia, CA, USA) and 2 μg total RNA was reverse transcribed to complementary cDNAs using Superscript II according to manufacturer's instructions (Bio‐Rad, Hercules, CA, USA). Specific primer sets were used: *GJA1* (CX43) forward 5′‐CAATCTCTCATGTGCGCTTCT‐3′ and reverse 5′‐GGCAACCTTGAGTTCTTCCTCT‐3′; *GJA5* (CX40) forward 5′‐CCGTGGTAGGCAAGGTCTG‐3′ and reverse 5′‐ATCACACCGGAAATCAGCCTG‐3′; *GJC1* (CX45) forward 5′‐ AGCTGTAGGAGGAGAATCCATC‐3′ and reverse 5′‐TGCAAACGCATCATAACAGACA‐3′; *GAPDH* forward 5′‐GGAGCGAGATCCCTCCAAAAT‐3′ and reverse 5′‐GGCTGTTGTCATACTTCTCATGG‐3′. Triplicate PCR reactions were performed using SYBR Green dye‐based detection method with a PCR Master Mix assay (Applied Biosystems, Waltham, MA, USA). A series of dilutions of cDNA from pooled samples were used to optimize the standard curve and validate the melting curves for each gene. GAPDH was used as a housekeeping gene for the normalization.

### 2.3 Immunoblotting

For the protein analysis of connexin protein levels and connexin 43 phosphorylation, the harvested tissues were homogenized and lysed in 10 mmol/L HEPES, pH 7.9, 1.5 mmol/L MgCl_2_, 10 mmol/L KCl, 1% NP40, and 1% sodium deoxycholate in the presence of protease and phosphatase inhibitors. Proteins were measured and the same amounts of proteins were then subjected to sodium dodecyl sulfate polyacrylamide gel electrophoresis, followed by electric transfer into polyvinylidene difluoride (PVDF) membrane. The antibodies used in the western blot included anti‐CX40 (Rabbit, 1:400; Millipore, Billerica, MA, USA), anti‐CX43 (Rabbit, 1:500; Cell Signaling, Danvers, MA, USA), anti‐phosphor‐CX43 (Rabbit, 1:300; Sigma, St. Louis, MO, USA), anti‐CX45 (Rabbit, 1:500; Abcam, Cambridge, MA, USA), and anti‐GAPDH (Rabbit, 1:800; Sigma). Signals were visualized using HRP‐conjugated secondary antibodies (Bio‐Rad) for ECL detection. The relative quantitation of protein bands was analyzed in ImageJ by using GAPDH as loading control.

### 2.4 Kinase assays

To measure the activities of PKA (protein kinase A) and PKC (protein kinase C), we used the kinase activity assay kits (Abcam) for individual targets. Based on the instructions of the kits, solid‐phase enzyme‐linked immunosorbent assays were used for the respective kinase target by incubating with their specific synthetic peptides as substrates. The resulted phosphorylated peptide was then recognized with a polyclonal antibody and measured by a HRP‐based detection method. For MAPK (mitogen‐activated protein kinase) pathway, an assay kit for ERK1/2 phosphorylation (Abcam) was used. In brief, the protein samples were incubated with phosphor‐specific capture antibodies and following detection antibodies. The signal was generated by HRP reaction and absorbance was measured at 450 nm.

### 2.5 Statistics

For human studies, each sample was aliquoted and repeated in triplicates for each analysis. The data were presented as the means ± *SD* as indicated, and the statistical comparison was analyzed by one‐way analysis of variance followed with a Tukey's post hoc test, if appropriate, with *p <* *.05 and **p <* *.01. For correlation analysis, we calculated Pearson's correlations between individual samples and their GCS scores. All analyses in the human cohorts were performed in SPSS statistical software with significance denoted as *p *<* *.05.

## 3 RESULTS

**Figure 1:**
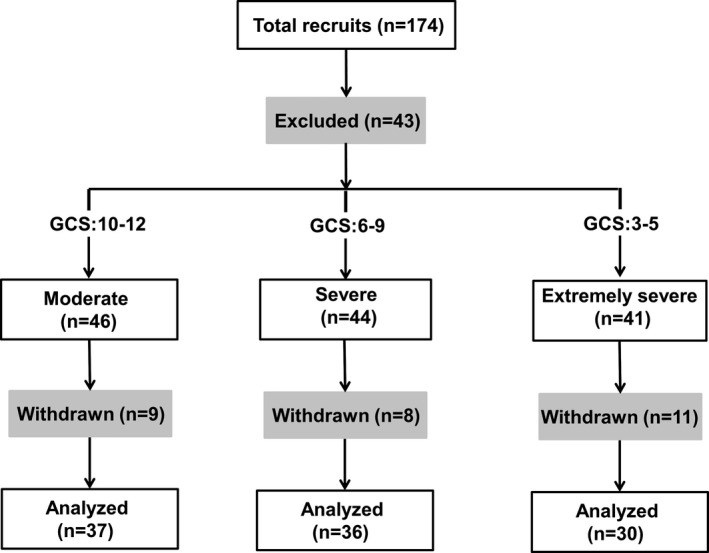
Experimental diagram in this study

We have evaluated total of 174 TBI patients according to inclusion/exclusion criteria for the study (see Materials and Methods). Recruited patients who have surgery needs (43 subjects excluded, Figure 1) were then categorized into three groups based on the Glasgow Coma Scale (GCS) scores as the indicator of brain injury extent. These groups were named as moderate (GCS 10–12), severe (GCS 6–9), or extremely severe (GCS 3–5). The baseline clinical characteristics of the patients who successfully completed studies (total 103) are shown in Table 1. The gender, age, education, and other general medical information of the patients are almost identical between patient groups except the duration of hospitalization. Of note, more than 70% of injuries involved accidents in all groups, which is consistent with previously reported etiologic data on TBI (Bruns & Hauser, 2003; Thurman, 2016). The injured tissue samples were collected on subjects during the brain surgeries. The following analyses included RNA, protein, and kinase activity measurements that were carried out on the harvested tissues.

**Table 1:** General characteristics of participants in the experimental groups

Characteristics	Moderate (*n* = 37)	Severe (*n* = 36)	Extremely severe (*n* = 30)
Male/female	21/16	23/13	17/13
Age (years)	37.2 ± 14.7	35.1 ± 13.4	38.3 ± 15.6
Education (years)	9.1 ± 2.5	8.8 ± 2.6	9.2 ± 2.8
Hospitalization (days)	16.6 ± 4.1	30.1 ± 7.6	52.3 ± 10.4
Hypertension	11 (29.7%)	12 (33.3%)	9 (30.0%)
Diabetes mellitus	4 (10.8%)	4 (11.1%)	3 (10.0%)
History of cardiovascular disease	3 (8.1%)	4 (11.1%)	4 (13.3%)
Etiological factors			
Accident	29 (78.4%)	27 (75.0%)	23 (76.7%)
Falling	4 (10.8%)	5 (13.9%)	4 (13.3%)
Others	4 (10.8%)	4 (11.1%)	3 (10.0%)
GCS	11.1	7.6	3.9

Data were given as mean ± *SD*.

GCS, Glasgow Coma Scale.

### 3.1 Expression levels of CXs are differentially altered in TBI

**Figure 2:**
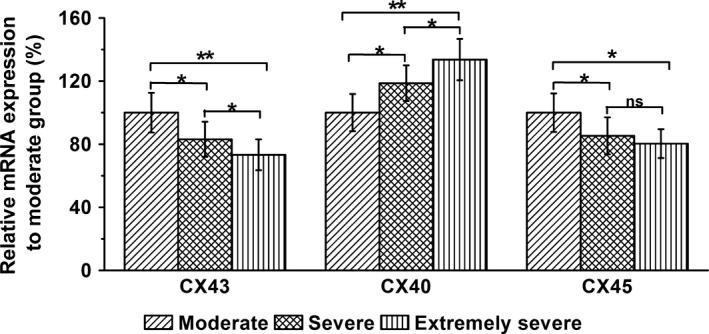
Relative mRNA expressions of CX43, CX40, and CX45 in the harvested brain tissues in three experimental groups (moderate, severe, and extremely severe groups). mRNA expressions were analyzed by RT‐PCR. GAPDH served as an internal control. Data were given as mean ± *SD*. *p < .05 and **p < .01. ns indicates no statistical difference

**Figure 3:**
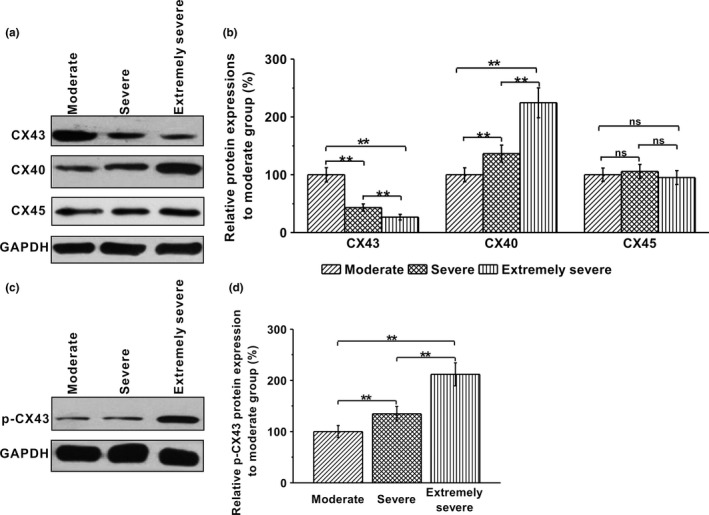
Protein expressions of CX43, CX40, and CX45, and CX43 phosphorylation in the harvested brain tissues in three experimental groups (moderate, severe, and extremely severe groups). (a, b) Representative protein bands for CX43, CX40, and CX45 and relative expressions to moderate group. Protein expressions were analyzed by western blotting. GAPDH was used as control. (c, d) Representative protein bands for p‐CX43 and relative expressions to moderate group. Data were given as mean ± *SD*. **p < .01. ns indicates no statistical difference

Results for mRNA analysis of CXs are shown in Figure 2. The levels of CX43 and CX45 (*GJC1*, gap junction protein gamma 1 or connexin 45) were moderately but significantly decreased in the severe TBI groups (severe or extremely severe patients), comparing to the levels of moderate patients (CX43: *p *= .033 and .007; CX45: *p* = .039 and .018 in severe and extremely severe groups, respectively). On the contrary, CX40 (*GJA5*, gap junction protein alpha 5 or connexin 40) mRNA level on the brain tissues rose following the increasing severity of the injuries (*p* = .024 and .003 in severe and extremely severe groups, respectively). We further performed western blots on these samples. As in the mRNA measurements, protein levels of CX43 and CX40 were reciprocally decreased and increased in severe TBI patients, respectively (Figure 3a, b; CX43: *p* = .004 and .002; CX40: *p* = .009 and .003 in severe and extremely severe groups, respectively). In contrast, there was no significant alteration of CX45 protein between the patient groups (Figure 3a, b; *p* = .374 and .492 in severe and extremely severe groups, respectively). Remarkably, phosphorylation of CX43, a major form of connexin involved in the pathogenesis of TBI (Frantseva et al., 2002), was enhanced in the severe patients, compared to the moderate subjects (Figure 3c, d; *p* = .007 and .006 in severe and extremely severe groups, respectively).

### 3.2 Comparison of potential CX43 phosphorylation activators in TBI patients

**Figure 4:**
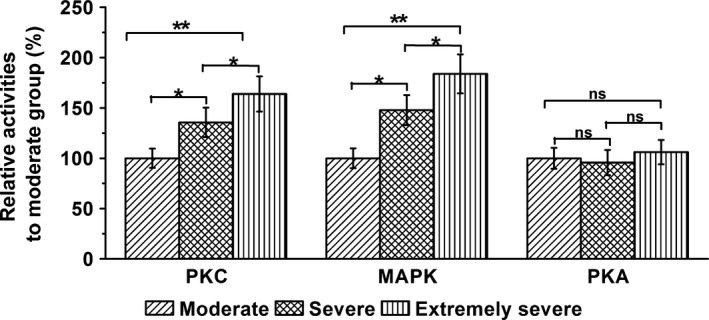
Relative activities of protein kinase C (PKC), mitogen‐activated protein kinase (MAPK), and protein kinase A (PKA) in three experimental groups (moderate, severe, and extremely severe groups) were examined by coimmunoprecipitation. Data were given as mean ± *SD*. *p < .05 and **p < .01. ns indicates no statistical significance

CX43 phosphorylation is known to be activated by protein kinases such as PKA, PKC, and MAPK (Schulz et al., 2015). Thus, we examined the relative activities of these modulators between the selected TBI patient groups. No significant change in PKA activity was found within the three patient groups (*p* = .781 and .396 in severe and extremely severe groups, respectively). As a comparison, the activity levels of PKC and MAPK were greatly enhanced in the injured brain tissues when the consciousness was severely impaired in the patients (Figure 4; PKC: *p* = .042 and .005; MAPK: *p* = .031 and .002 in severe and extremely severe groups, respectively).

### 3.3 Relation of CXs with the severity of TBI

**Figure 5:**
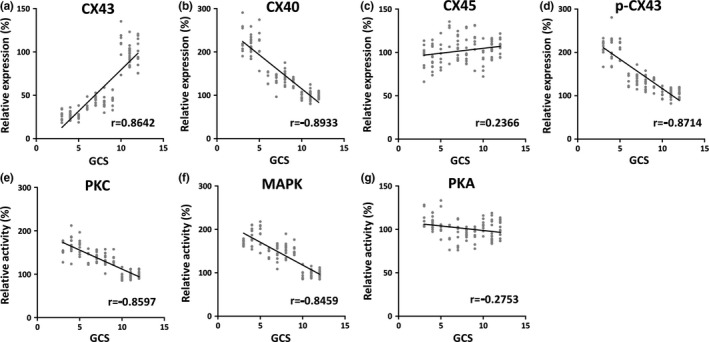
Correlation studies between Glasgow Coma Scale (GCS) and CX43 protein level (a, *r* = .8642), CX40 protein level (b, *r* = −.8933), CX45 protein level (c, *r* = .2366), p‐CX43 protein level (d, *r* = −.8714), protein kinase C (PKC) activity (e, *r* = −.8597), mitogen‐activated protein kinas (MAPK) activity (f, *r* = −.8459), and protein kinase A (PKA) activity (g, *r* = −.2753) in all participants (*n* = 131)

To further investigate the correlation between the above CXs or its regulators and the severity of TBI, we performed a series of Pearson's correlation analyses in all patient samples (Figure 5). As expected, the individuals with the lowest GCS score reflecting the most severe defects contained the lowest levels of CX43 protein (*p* = .003, *r* = .8642), the highest levels of CX40 protein (*p* = .002, *r* = −.8933) and CX43 phosphorylation (*p* = .007, *r* = −.8714), and accompanied by the enhanced activities of PKC (*p* = .009, *r* = −.8597) and MAPK (*p* = .007, *r* = −.8459). The correlations were highly significant. CX45 protein level (*p* = .273, *r* = .2366) and PKA activity (*p* = .174, *r* = −.2753), on the other hand, failed to correlate with the GCS score (Figure 5c, g).

## 4 DISCUSSION

In summary, we investigated the connexin family proteins for the first time in the human brain tissues of TBI patients. Three CXs studied in the current study (CX43, CX40, and CX45) were detected and some of their mRNAs/proteins changed with the severity categories. It was highlighted in our studies that the severity of TBI based on the GCS is associated with the levels of both CX43 and CX40 proteins, along with the protein phosphorylation status of CX43. Follow‐up experiments have provided evidence that the putative kinase activators of CX43 phosphorylation such as PKC and MAPK also correlate with the progression of brain damage in the TBI individuals. Taken together, our data in human patient are consistent with the previous association of CXs in animal models of brain injury, and suggest that several related molecules, including CX43, CX40, and phosphorylated CX43, could be important contributors for the pathogenesis of TBI.

TBI caused by physical accidents is an increasing concern and serious health problem that can lead into a variety of CNS symptoms including concussion, brain edema, and unconsciousness. Given the unclear mechanism and the lack of consensus regarding the assessment of cognitive dysfunction due to TBI (Zheng & Tong, 2015), molecular changes responsible for the neuropathological abnormalities in TBI patients are of particular clinical interest. CXs are known to be modulated during the neuronal injuries in animals, with strong associations with the deficits in cognitive or behavioral functions after the injuries. To test the role of CXs in brain damage, we took an early step in studying CXs directly on human tissues collected from TBI patients. Various locations of brain injuries were included in the study and extents were also evaluated in patients by computerized tomography (CT) scans. Unlike animal models in which control or sham‐operated individuals were often included in comparison, all subjects in the current study have significant brain damages because of the surgical requirement for harvesting brain tissues. The normal patient controls or patients with mild symptoms who do not need the brain surgery were excluded from the study. Nevertheless, the development of strong correlation between CX40/CX43 levels of the tissue samples and GCS scores of TBI patients clearly point to the importance of CXs as critical players involved in brain injuries. In addition, metabolic conditions like diabetes and hypertension were indistinguishable between patient groups, minimizing potential confounding effects from other known factors on connexin expression (Figueroa, Isakson, & Duling, 2006; Hamelin, Allagnat, Haefliger, & Meda, 2009; Rummery & Hill, 2004; Wright, Richards, & Becker, 2012).

Connexins may be structural components in rearranging intercellular communications in response to injury‐induced CNS remodeling. To maintain cell viability and reestablish disrupted synaptic connections following the neuronal injury (Chang, Pereda, Pinter, & Balice‐Gordon, 2000; Yu et al., 2014), connexin expressions could be beneficial to TBI. On the other hand, changes in connexin levels may exacerbate brain dysfunctions (Fonseca, Green, & Nicholson, 2002). It was previously reported by in vitro and in vivo models of TBI and epilepsy, neuronal gap junction coupling through CX36 can rise along with ischemia‐stimulated neuron death that is regulated by glutamate‐mediated mechanisms (Wang et al., 2012). CX43 mRNAs, which are primarily seen in astrocytes in the rat, were significantly enhanced following spinal cord injury (Lee, Lindqvist, Kiehn, Widenfalk, & Olson, 2005). An upregulation of CX40 was discovered in a mouse model of TBI‐induced brain injury as early as 6 hr postinjury (Chen et al., 2015). In a rat model of fluid‐percussion TBI, CX40 protein increased, while CX45 protein expression decreased at the cerebral arteries 24 hr after injury (Avila et al., 2011). Consistent with all these data, the current human study suggested the involvement of connexin proteins in the pathogenesis of TBI. Although the accessibility of the injured tissue is limited and a follow‐up study of the patient outcomes is needed, the correlations found here are significant. For instance, individuals in the top quantile of CX43 protein had approximately twofold extent of consciousness deficit with those in the lowest quartile. A future assessment of the results in a large cohort will help to extend the study in which a noninvasive approach is required for case–control analyses. An important strength of the investigation also lies on the examination of CX43 phosphorylation.

The phosphorylation of CX43 at the hippocampus and cortex was significantly increased at rats following brain injuries (Ohsumi et al., 2010; Sun et al., 2014). The stimulated phosphorylated CX43 also colocalizes with autophagy markers in the hippocampal neurons following TBI (Sun et al., 2014), suggesting astrocytic gap junction coupling could be associated with autophagy that is a critical regulator in various forms of brain injury. Both PKC and MAPK are activated in the settings of brain injuries and represent as putative therapeutic targets in the treatment of experimental TBI (Lu, Cheng, Wu, & Yang, 2008; McIntosh, 1993). Interestingly, they can directly phosphorylate CX43 in response to various stimuli (Schulz et al., 2015). Indeed, we found that activities of PKC and MAPK in the human samples were strongly associated with TBI severity, consistent with a stimulated CX43 phosphorylation pathway as the occurrence of exacerbated deficits in cognition. With the availability of more phosphor antibodies to various forms of CXs, we predict the human study of connexin profiling may further boost the identification of novel markers for neurological disorders.

## 5 CONCLUSION

In conclusion, our findings suggest that CXs at the injury sites are correlated with TBI severity and they could be essential modulators of neuronal integrity in humans. The association of TBI severity with phosphorylated CX43 and PKC/MAPK also potentially support a specific pathway involved in brain damage in TBI patient. Finally, the results of the current study may prompt an interest in studying specific CXs to delineate severity of TBI.

## CONFLICT OF INTEREST

The authors declare that they have no conflict of interest.
